# Effect of Remdesivir on mortality and length of stay in hospitalized COVID-19 patients: A single center study

**DOI:** 10.12669/pjms.38.ICON-2022.5779

**Published:** 2022-01

**Authors:** Quratulain Shaikh, Samreen Sarfaraz, Anum Rahim, Mujahid Hussain, Rabeea Shah, Sara Soomro

**Affiliations:** 1 Dr. Quratulain Shaikh, FCPS, MSc, Department of Internal Medicine, The Indus Hospital and Health Network, Karachi, Pakistan; 2 Dr. Samreen Sarfaraz, MRCP, Department of Internal Medicine, The Indus Hospital and Health Network, Karachi, Pakistan; 3 Dr. Anum Rahim, MBBS, MSc. Indus Hospital Research Center, The Indus Hospital and Health Network, Karachi, Pakistan; 4 Mujahid Hussain, PharmD, MSc (Clinical Pharmacy), Department of Pharmacy Services, The Indus Hospital and Health Network, Karachi, Pakistan; 5 Dr. Rabeea Shah, FCPS, Department of Internal Medicine, The Indus Hospital and Health Network, Karachi, Pakistan; 6 Dr. Sara Soomro, MBBS, Department of Internal Medicine, The Indus Hospital and Health Network, Karachi, Pakistan

**Keywords:** COVID-19, Remdesivir, mortality, ARDS

## Abstract

**Objectives:**

To see the difference in mortality among hospitalized COVID-19 patients given Remdesivir (RDV) with those who were not given RDV.

**Methods:**

A prospective cohort study was conducted on patients who were admitted to the COVID-19 isolation unit at The Indus Hospital, Korangi Campus Karachi between March and June 2020.

**Results:**

Groups were similar in age and gender distribution. RDV group was more hypoxic, had severe ARDS and needed higher Oxygen support compared to non-RDV group (p=0.000). Median SOFA score was 2 in RDV vs 5 in non-RDV (p=0.000). More than moderate COVID pneumonia was found in 92% of the RDV group while 89% of non-RDV group (p value=0.001). Median day of illness to administer Remdesivir was 10. There was no difference in mortality (45.5% in RDV vs 40.4% in non-RDV; p=0.4) between the two groups. Median length of hospital stay was 12 days (IQR=7.5-14.5) in RDV group compared to 10 days (IQR=6-14) in non-RDV group (p=0.009).

**Conclusion:**

RDV did not show any difference in in-hospital mortality in our patients. More patients had severe ARDS in the RDV group while patients in the non-RDV group had higher SOFA score and multi-organ failure. Length of stay was longer in patients receiving Remdesivir.

## INTRODUCTION

The novel coronavirus disease (COVID-19) emerged in December 2019 and rapidly became a pandemic. Globally, as of now 203,178,675 people are infected, including four million deaths.^1^ This led to the search of desperate public health measures including the quest for effective treatments. Despite the fact that a number of therapeutic agents have been tested for the treatment of Covid-19, no antiviral drug has proved to be efficacious.^2^ Experts from the World Health Organization initiated mortality trials comparing four drugs including Remdesivir, Hydroxychloroquine, Lopinavir, and interferon beta-1a.^3^ The rate of mortality, initiation of ventilation, and length of hospital stay were not definitely reduced by any of the trial drug.^3^

One of the many promising candidates for this purpose was Remdesivir, which is a drug with effective antiviral properties.^4^ Remdesivir has a broad spectrum of activity and acts by inhibiting RNA-dependent RNA polymerases (RdRp) and subsequently causes arrest in RNA synthesis.^5^ It has been used to treat Filo viruses, Corona viruses, paramyxoviruses and pneumoviridae.^5^ In Wuhan, China the first randomized, placebo-controlled trial of Remdesivir among patients with COVID-19 was begun. Unfortunately, they were unable to access the efficacy of Remdesivir as the trial was unable to successfully complete recruitment.^6^ However, results from a large randomized, double-blind clinical trial showed that a 10-day Remdesivir course had significantly faster recovery time (11 days) in COVID 19 patients when compared to those who received placebo (15 days).^2^ Contradicting this evidence, the WHO led SOLIDARITY trial in over 40 countries worldwide showed no benefit of Remdesivir on mortality or length of hospital stay.^3^ Despite such conflicting results, on 1^st^ May 2020, Remdesivir was granted Emergency Use Authorization by the US Food and Drug Administration for the treatment of patients with severe COVID-19.^7^ Later this was expanded to include non-severe patients. Final approval was given on the basis of three Randomized controlled trials which showed benefit of Remdesivir over placebo in reducing mortality.^2^,^4^,^8^ Hundred and Eight clinical trials of Remdesivir for COVID-19 are currently registered with U.S National Library of Medicine.^9^ The only contraindications to the use of Remdesivir are severe hepatic dysfunction (ALT >5 times of upper limit of normal), renal dysfunction with GFR <30 ml/min and neonates.

Currently, Pakistan has entered the fourth wave of COVID-19. So far, there have been about 21 thousand deaths due to COVID-19 all over the country.^10^ It is extremely crucial to aim for effective therapeutic regimes for COVID-19 along with prevention strategies. In this paper, we share our experience with Remdesivir use in COVID-19 hospitalized patients at the Indus Hospital, Korangi Campus, Karachi during the first wave of COVID-19 in the country.

## METHODS

We conducted a prospective cohort study on all COVID-19 admissions (positive nasopharyngeal RT-PCR for COVID-19) at the Indus Hospital, Korangi campus in Karachi. The study was approved by the IRB under approval number IRD_IRB_2020_04_002. All consecutive patients who were admitted to the COVID-19 isolation unit and were at least 18 years old were enrolled between 24th March and 19th June 2020. Disease severity was assessed using the National COVID-19 management guidelines 11. Disease severity was classified as “severe COVID-19” if on admission the respiratory rate was more than 30/min, oxygen saturation was 90% on room air or Chest X-ray showed more than 50% of lung fields involved while “Critical COVID-19” if along with the criteria for severe disease they manifested ARDS, Septic shock or multisystem organ failure (MSOF). Remdesivir was administered to patients who fulfilled the criteria for severe disease as per the international recommendations at that time. Since the drug was not easily available in Pakistan at that time, the choice of patients for therapy was largely dependent on availability of the drug. Later, Remdesivir was being manufactured in Pakistan and was easily available. Only contraindications to the use of Remdesivir were pre-existing hepatic dysfunction as manifested by Alanine Aminotransferase (ALT) levels more than 5 times the upper limit of normal and reduced Glomerular filtration rate (<60 ml/min). Hence, those COVID-19 severe disease patients who received Remdesivir were analyzed in Rem group while those who could not receive Remdesivir due to unavailability or contra-indications were analyzed in Non-Rem group. Those patients who did not receive Remdesivir due to unavailability of the drug in the early days or any contraindication to its use were used as a comparison group for the purpose of this analysis. We recorded patient’s demographics, clinical details and laboratory parameters at admission. The main outcome of interest was in-hospital mortality. All data was entered on a REDCap database and Stata version 14 was used for analysis. Mean (SD) were reported for continuous data after checking normality. Median (IQR) was used for non-normally distributed data. Categorical variables were reported as numbers and percentages. Either Student’s t-test or Mann Whitney U test were used to compare continuous data. Chi square or Fischer’s Exact test was used to compare categorical data. P-value ≤ 0.05 was considered significant.

## RESULTS

A total of 268 patients were enrolled in the study. Remdesivir was given to 102 patients while 166 were in the non-Remdesivir group. The baseline characteristics of both Remdesivir and non-Remdesivir groups are shown in Table-I.

**Table I:** Characteristics of COVID-19 patients admitted at The Indus Hospital-Korangi Campus.

Variable	Remdesivir n(%) n=102	Non-Remdesivir n(%) n=166	p-value
Age^β^	55.0 ± 12.8	56.2 ± 13.3	0.485
**Gender**			
Male	75 (73.5)	130(78.3)	0.370
Female	27(26.5)	36(21.7)	
**Hypertension**			
Yes	47(46.1)	72(43.4)	0.665
No	55(53.9)	94(56.6)	
**Diabetes**			
Yes	52(51.0)	74(44.6)	0.308
No	50(49.0)	92(55.4)	
**Heart Disease**			
Yes	11(10.8)	17(10.2)	0.888
No	91(89.2)	149(89.8)	
**Fever**			
Yes	86(84.3)	139(83.7)	0.900
No	16(15.7)	27(16.3)	
**Cough**			
Yes	51(50.0)	101(60.8)	0.089
No	51(50.0)	65(39.2)	
**Shortness of breath**			
Yes	76(74.5)	139(83.7)	0.066
No	26(25.5)	27(16.3)	
Systolic BP^ mmHg	138(118-151)	139(123-153)	0.437
Diastolic BP^ mmHg	80(71-80)	80(72-90.5)	0.847
Pulse^ b/min	100(86-110.5)	106(92-117.5)	0.233
Respiratory Rate^ /min	32(26-38)	30(24-36)	0.147
Oxygen Saturation^ %	86(78-90)	88(76-93)	0.032*
SOFA Score^	2(2-3.5)	5(4-8)	<0.0001**
PaO2/FiO2 Ratio^	115(72.9-242.5)	187.5(104.3-268.3)	0.032*
**Clinical Severity**			
Moderate	8(7.8)	18(10.8)	0.001**
Severe	27(26.5)	15(9.0)	
Critical	67(65.7)	133(80.1)	
**Invasive ventilation**			
Yes	37(36.3)	66(39.8)	0.569
No	65(63.7)	100(60.2)	
**ARDS**			
Severe ARDS	41(40.2)	30(19.2)	0.002*
Moderate ARDS	20(19.6)	52(33.3)	
Mild ARDS	24(23.5)	46(29.5)	
Not Present	17(16.7)	28(17.9)	
**Oxygen Requirement**			
≥5 Litres	83(81.4)	84(50.6)	<0.0001**
? 5 Litres	19(18.6)	82(49.4)	
**Non Invasive Ventilation**			
Yes	39(38.2)	63(38.0)	0.963
No	63(61.8)	103(62.0)	

^Median (IQR),

β Mean (SD), P value

* <0.05,

** <0.001

Overall more than three fourth (76.5%) of the patients were male and there was no difference in gender or age distribution of the two groups. Distribution of major comorbid conditions like diabetes mellitus, hypertension and heart disease was not different between the two groups. Most patients presented with fever, cough and shortness of breath as presenting symptoms in both groups (p>0.05). Among the physical parameters in emergency room, median peripheral oxygen saturation (Spo2) was significantly lower in the Remdesivir group (86% vs 88%; p=0.032). Similarly, the median Pao2/Fio2 ratio was much lower for the Remdesivir group (115; IQR=72.9-242.5) at presentation compared to the non-Remdesivir group (187; IQR=104-268) (p=0.032). In the Remdesivir group, 40% patients had severe ARDS while in the non-Remdesivir group only 19% had severe ARDS (p=0.002). Patients with clinical severity of moderate, severe and critical were included in the study as those with less than moderate disease were not admitted to hospital for management. Remdesivir group had more patients (92%) with combined severe disease and critical disease while the non-Remdesivir group had a majority of patients with critical disease (80%) (p-value=0.001). Need for high flow oxygen at least 5 L was more in the Remdesivir group (81% vs 50.6%; p=0.000). However, both the groups had similar need for invasive ventilation and non-invasive ventilation (p>0.05). Median SOFA score was much higher in non-Remdesivir group depicting multiorgan dysfunction compared to the Remdesivir group (5 vs 2; p=0.000).

There were no major differences in baseline laboratory parameters (Table-II), however, inflammatory markers like Ferritin and IL-6 were markedly raised in the non-Remdesivir group compared to Remdesivir group (p<0.05). Median D-dimer was 0.6 ng/ml in Remdesivir group while 1.8 ng/ml in the non-Remdesivir group (p=0.000). Pro-calcitonin was significantly higher in the non-Remdesivir group (p=0.013). Chest X-ray findings were scored using RALE scoring and the median RALE score was 6 (IQR=5-8) in Remdesivir group while 5 (IQR=4-7) in the non Remdesivir group (p value 0.002).

**Table II:** Baseline Laboratory parameters.

Variable	Remdesivir	Non-Remdesivir	p-value

	Median (IQR)	Median (IQR)	
Hemoglobin (mg/dl)	12.8(11.8-14.1)	13.2(11.4-14.5)	0.921
White blood cells (x10^9^/L)	15.6(9.5-17.8)	12.5(7.9-17.5)	0.883
Neutrophils (%)	87.6(82-90.9)	85.3(80.3-90.2)	0.809
Lymphocytes (%)	6.3(5.4-11.6)	8.2(7.1-12.8)	0.924
Platelet (x10^9^/L)	293(106.5-367.5)	202(131-341)	0.227
Creatinine (mg/dl)	1.0(0.8-1.3)	1.1(0.9-1.9)	0.018*
IL6 (pg/ml)	24.2(10.2-50.5)	46.3(24.8-164.8)	0.059
CRP (mg/L)	151.7(47.7-206.8)	135.7(77.1-214.9)	0.401
Ferritin (ng/ml)	730.1(393.7-1076.5)	1623(606.9-1675.6)	<0.0001**
Pro-calcitonin (ng/ml)	0.2(0.1-0.4)	0.6(0.2-2.2)	0.013*
LDH (U/L)	423.5(341.3-579.3)	529.5(453.8-767)	0.772
D-dimer (ng/ml)	0.6(0.4-1.9)	1.8(0.7-6.9)	<0.0001**
RALE Score (CXR)	6(5-8)	5(4-7)	0.002*

CXR= chest x-ray, P value

* <0.05,

** <0.001

When mortality was compared between the two groups (Table-III), no major difference in hospital mortality was observed (46% in Remdesivir group vs 40.4% in non-Remdesivir group; p=0.46). Median length of hospital stay was 12 (IQR=7.5-14.5) days compared to 10 days (IQR=6-14) in non-Remdesivir group (p=0.009).

**Table III:** Outcome COVID_19 patients by use of Remdesivir.

	Remdesivir	Non-Remdesivir	p-value
**In-hospital complications** ^β^	n(%)	n(%)	
None	36(38.3)	78(50.0)	<0.0001**
Cardiac Abnormalities	22(23.4)	27(17.3)	
Nosocomial Infection	16(17.0)	30(19.2)	
CNS Abnormalities	3(3.2)	5(3.2)	
Septic Shock	6(6.4)	32(20.5)	
MODS	15(16.0)	23(14.7)	
AKI	10(10.6)	46(29.5)	
Thromboembolism	0	7(4.5)	
Barotrauma	0	4(2.6)	
DIC	1(1.1)	9(5.8)	
**Length of hospital stay^(days)**	12(7.5-14.5)	10(6-14)	0.009*
**Mortality**			
Alive	55(53.9)	99(59.6)	0.406
Dead	47(46.0)	67(40.4)	

^ Median (IQR),

β does not add up to 100, P value

* <0.05,

** <0.001

With regards to adjunctive therapies, (Table-IV) there was no significant difference in the use of Tocilizumab between the two groups (p>0.05). Use of antibiotics was higher in non-Remdesivir group (p=0.000) while use of methyl prednisolone was higher in Remdesivir group (p=0.001). Similarly, the use of therapeutic anticoagulation was significantly higher in the non-Remdesivir group compared to Remdesivir group (p=0.001).

**Table IV:** Use of adjunct therapies in both groups of COVID-19 patients.

Variable	Remdesivir n(%)	Non-Remdesivir n(%)	p-value
**Tocilizumab**			
Single Dose	25(25.3)	34(20.5)	0.615
Two Doses	4(4.0)	9(5.4)	
Not Given	70(70.7)	123(74.1)	
**Antibiotics**			
Yes	46(46.0)	142(86.6)	<0.0001**
No	54(54.0)	22(13.4)	
**Anticoagulation**			
Therapeutic Doses	29(29.0)	77(47.2)	0.001**
Prophylactic Doses	68(68.0)	73(44.8)	
Not Given	3(3.0)	13(8.0)	
**Methylprednisolone**			
Yes	97(97.0)	135(82.8)	0.001**
No	3(3.0)	28(17.2)	

The median number of doses used for this cohort was 5 (IQR=2-5), which corresponds to 600mg Remdesivir per patient. The median duration of symptoms at which the Remdesivir was administered was 10 days (IQR=8-11). The maximum Alanine amino transferase after administration of Remdesivir was 81 (IQR=42-136) wile maximum creatinine was 1.1 (0.9-2.2) in our patients.

## DISCUSSION

We believe this is the first report of the use of Remdesivir from our country. Randomized Clinical Trials have shown some benefit of Remdesivir in COVID- 19 pneumonia.^2^,^4^,^8^ However, our data is from the first wave of COVID-9, when practitioners did not have much idea regarding the optimum dosing and duration of therapy as well as expected side effects of this uncommon drug. With more data emerging on the management of COVID-19, it is now evident that Remdesivir is one of the mainstay therapies for halting the progression of COVID-19 pneumonia.

Although our data shows no effect of Remdesivir on mortality, we have only been able to look at in-hospital mortality while a recent meta-analysis published in January 2021 suggests that 28 day mortality and oxygen support through 14-28 days is affected by the use of Remdesivir.^12^ Hence, it is quite possible that our groups would have shown a difference if they were followed for a longer period of time. Secondly, our treatment groups were not balanced to begin with. The Remdesivir group was characterized by marked hypoxia and features of ARDS due to COVID-19 pneumonia allowing easy decision making for using the drug. On the other hand, the non-Remdesivir group seems to be more severely sick as indicated by higher median SOFA scores and high pro-calcitonin suggesting the onset of multi organ dysfunction and possible secondary bacterial infection complicating their outcomes and prognosis. The use of Remdesivir was abandoned in this group due to presence of multi organ dysfunction and probable liver and renal injury contraindicating the use of Remdesivir. However, the overall mortality reported from another one of our recently published papers from this cohort was 39% which was not very different from the Remdesivir group here.^13^

One important difference to highlight in our data is that Remdesivir was being administered to people at a median 10th day (IQR=8-11) of illness during the first wave of COVID-19. Later, published data indicated that this was probably already late. Hence, guidelines adapted the 10 day rule and now it is understood that Remdesivir benefit is maximized by giving it in hypoxic patients within 10 days of illness.^11^ In fact, some data suggests the use of Remdesivir in non-hypoxic patients earlier in the course of the disease if there is evidence of COVID-19 pneumonia.^14^ We did not identify any major side effects of Remdesivir in our patients. Maximum ALT and serum creatinine were not significantly raised.

Our study has a number of limitations including non-random treatment allocation leading to selection bias. Co-administration of other compassionate therapies like antibiotics, steroids and anticoagulation (both prophylactic as well as therapeutic doses) could also confound the results. These effects are not easy to tease out because of the observational nature of the study. An interesting observation was that most patients in the non-Remdesivir group were given antibiotics, had higher pro-calcitonin levels and higher SOFA scores indicating the onset of secondary bacterial infection or an already complicated clinical course with multi-organ failure. Hence this group, was never a candidate for Remdesivir therapy. While most patients in the Remdesivir group had pure hypoxia, need for high flow oxygen and severe ARDS without evidence of multi-organ failure as evidence by a lower SOFA score. Even in this group, we see a high mortality and no benefit of Remdesivir in our patients. We believe this group of patients were too late to receive Remdesivir, as has been discovered later in multiple studies, hence we were not able to see any improvement in outcomes.^14^ However, the study is unique in being one of the first local reports on Remdesivir from our region where prospective data collection was done to follow patients for in-hospital mortality and side effects. Our recent data on the use of Remdesivir in non-hypoxic, moderate COVID pneumonia patients is soon to be published showing significant difference by the use of Remdesivir. It is in the light of this data that our institution’s guidelines were modified to include the use of Remdesivir in moderate COVID-19 pneumonia patients (unpublished).

Although our results are not encouraging in favour of the use of Remdesivir but due to the limitations highlighted above we believe further randomized controlled trials and prospective data on early administration of Remdesivir in COVID-19 pneumonia are needed to provide evidence of effect. Moreover, longer follow up studies on long term outcomes and safety are needed. COVID-19 is currently the biggest healthcare problem and we still do not have enough effective therapies for this disease. Hence, the search for better therapeutic modalities and optimum timing of therapy is important in management of COVID-19 patients.

### Conclusion:

Remdesivir did not show any mortality benefit among severe COVID-19 patients in our data. Randomized controlled trials are needed to define the effectiveness of the drug early in the course of the disease.

### Authors’ Contributions:

**QS:** Conceived, designed, data collection, data analysis, manuscript writing.

**SS:** Conceived, designed, data collection, manuscript review.

**AR:** Designed, data collection, data analysis, results writing.

**MH, RS & SS :** Designed, data collection, manuscript review.
